# Understanding the challenges, opportunities, and drivers to addressing health inequalities within local health systems: the UNFAIR case study qualitative project

**DOI:** 10.1186/s12913-025-12956-7

**Published:** 2025-06-06

**Authors:** Lorraine McSweeney, Joanne Lally, Charlotte Parbery-Clark, Kylie Murrell, Richard G. Thomson, Sarah Sowden

**Affiliations:** 1 Population Health Sciences Institute, Newcastle University, Newcastle upon Tyne, UK; 2 Gateshead Council, Regent Street, Gateshead, UK

**Keywords:** Health inequalities, Health care systems, Case study methodology, Qualitative methods

## Abstract

**Background:**

Despite policy prominence and frameworks focusing on health inequalities, healthcare leaders do not feel they have the skills and knowledge to reduce health inequalities. This comparative case study explored four areas in England to understand the challenges, opportunities, and drivers of local health systems addressing health inequalities and what ‘good’ practice might look like.

**Methods:**

Interviews were held with 46 people working in health care services across the NHS, local authority or voluntary, community, social enterprise sectors. Key documents (*n* = ~ 10) in each of the four areas relating to reducing health inequalities were analysed using documentary analysis methods. Interviews and documents were coded and analysed independently before being integrated to synthesise findings. Analysis was conducted using a two-stage approach - firstly, an inductive analysis of emergent themes; secondly, to build knowledge on each case studys’ system approach of reduction of health inequalities, principles of the Action Scales Model were used.

**Results:**

Nineteen themes were identified across the four case studies; some themes were not apparent in all the case studies, nor in either the documentary analysis or interviews. These themes allowed us to compare between cases to explore what might be contributing to good practice. Themes identified included: understanding the local context; facilitators of how to tackle health inequalities and improve health and wellbeing; and future concerns. The secondary analysis highlighted potential levers for action from each case study; these included optimising retention and recruitment of workforce and allowing time and resources for longer-term planning. Two case study areas which appeared to have system resilience, demonstrated having a shared vision, strong partnerships, understanding of the system, and putting people and communities at the heart of decision making.

**Conclusion:**

This comparative case study makes a crucial contribution in the understanding of health systems addressing health inequalities in their local areas. The combined interview and documentary analysis findings provide rich insights of local systems’ documented strategies, plans and what is happening ‘on the ground’.

**Supplementary Information:**

The online version contains supplementary material available at 10.1186/s12913-025-12956-7.

## Background

Health inequalities refer to avoidable differences in health outcomes between groups or populations such as variations in life expectancy or the age at which preventable diseases occur. These disparities are closely linked to social, economic, and environmental factors [1]. The causes of health inequalities are complex but are generally related to a range of factors that positively or negatively influence a person’s ability to have good health [1]. A higher exposure to risk factors detrimental to health, such as smoking and poor diet, and/or lower access to preventative and health care services, can contribute to health inequalities [2], as can intersectionality, which can put certain groups at a significant disadvantage to seeking healthcare [3, 4]. Additionally, wider determinants of health such as access to education, work, income, deprivation, housing and social networks can also have an impact [1]. The recent COVID-19 pandemic has exacerbated existing health inequalities, particularly affecting lower socio-economic and minoritised ethnic groups [3, 5, 6]. In the UK, prior to COVID-19, health inequalities were estimated to cost the National Health Service (NHS) an extra £4.8 billion a year. In 2019, the NHS *Long Term Plan* outlined action to drive down health inequalities [7]. Chapter Two of the plan set out that ‘new funded action would strengthen the NHS’s contribution to prevention and health inequalities’ which would ‘establish a more concerted and systematic approach to reducing health inequalities’ [7]. Major reforms to the organisation of the NHS in 2022 established 42 integrated care systems (ICS) to plan and coordinate local services [8]. The premise being that cross-sector collaboration is needed to improve health and reduce health inequalities [8]. The health and care bill [9] was intended to put ICSs on a statutory footing with legal responsibilities of proactively reducing health inequalities and formalising relationships across a board of coalition of partners [10]. Whilst the ICSs came into fruition during the time of the current study, and therefore were included; they are only one part of the system. All the organisations as defined by our case studies can contribute to the health inequalities agenda and the way in which they do that is shaped by policy as well as local contexts.

However, the Long Term Plan and its subsequent supporting documents failed to outline how local and national systems could systematically approach health inequalities, with an expectation that local healthcare systems would each develop their own approaches [5]. It has been reported that, despite policy prominence and frameworks focusing on health inequalities, healthcare leaders do not feel they have the skills and knowledge to reduce health inequalities [5]. The UK Office for Health Improvement and Disparities (OHID) in 2022 produced guidance for addressing health inequalities [1]. This includes an emphasis that everyone, including health professionals, local authorities and voluntary, community and social enterprise (VCSE) organisations which have their own statutory responsibilities and accountabilities can contribute to reducing health inequalities in their everyday practice [1]. The wider determinants of health must also be considered. For example, local areas have a mandatory requirement to have a joint strategic needs assessment (JSNA) and joint health and wellbeing strategy (JHWS) whose purpose is to ‘improve the health and wellbeing of the local community and reduce inequalities for all ages’ [11]. This includes addressing the wider determinants of health [11]. There are myriad interventions and strategies in place, for example improving health literacy [1], proactively engaging those at risk of poor health outcomes in preventative programmes and supporting mental health [12] but there is scant literature on what ‘good’ looks like and how best to operationalise effective strategies.

To effectively deal with most public health challenges, including reducing health inequalities and improving population health, broader integrated approaches [13] and an emphasis on systems is required [14, 15]. A system is defined as ‘the set of actors, activities, and settings that are directly or indirectly perceived to have influence in or be affected by a given problem situation’ [16]. Local health systems are increasingly being tasked with driving action to reduce social inequalities in health. However, reorientating health systems towards prevention and the wider determinants of health is challenging [17]. Furthermore, the uptake of systems approaches to address public health challenges has been slow and remains largely theoretical [18]. Understanding what works and what does not work ‘on the ground’ is essential. Having a systems’ thinking focus around public health challenges can help support the evaluation of policy and programmes, and the development of interventions which recognise wider system influences [19]. The utilisation of a ‘systems’ thinking lens encourages the consideration of how systems (actors, activities and settings) relate to one another and how activities in one part of a system may affect another [16, 19]. Within systems theory, leverage points exist which are modifiable points within a system that, if altered, can lead to changes in how the system functions [18].

This study uses case study methodology to explore the challenges, opportunities, and drivers of four local health care systems in England in working to reduce health inequalities.

## Methods

### Study aim

To explore and understand the challenges, opportunities, and drivers of local health systems addressing health inequalities and what ‘good’ practice might look like.

### Study design

We adopted Yin’s multiple comparative case study approach [20], because this enables an in-depth investigation of issues, events or phenomena, in their natural real-life context across a number of local areas [21]. Case study methodology is increasingly being used in health research as it helps to answer ‘how’ and ‘why’ questions about complex issues in their natural setting [22]. Case studies comprised professionals working in health and social care sectors, for example from NHS, local authority, Public Health organisations and VCSE. Ethics approval was obtained by Newcastle University*’s* Ethics Committee (ref 13633/2020). A pilot case study was conducted to inform subsequent data collection and analysis methods of the remaining case studies [23].

### Study selection

Four case studies were purposively chosen to reflect a range of overall deprivation level of the area (Indices of Multiple Deprivation (IMD) [24]), their urban/rural location and differing geographical spread across the UK. Of consideration was a pragmatic judgement of likely ability to achieve the depth of insight required [20] and input from the project public involvement advisory panel.

### Semi-structured interviews

The key stakeholders were identified through the documentary analysis and in consultation with the research advisory group comprising experts in the field. Initially board level committee members (including lay, managerial, and clinical members) within relevant local organisations were identified. Snowball sampling [25] was undertaken whereby interviewees helped to identify additional key informants. Interview questions were based on an iteratively developed topic guide (Appendix 1), informed from previous work’s findings [26] and the study research advisory network’s input and a series of pilot interviews. A study information sheet was emailed to prospective interviewees, and participants were asked to complete an e-consent form using Microsoft Forms [25]. Interviewees were interviewed by LM or CPC using the online Teams platform and lasted up to one hour. Interviewees were asked a range of questions including any work relating to reducing health inequalities within the last five years. The interviews were recorded, using the built in Teams function, transcribed verbatim and anonymised.

### Case study documentary analysis

The documentary analysis (DA) followed the READ approach [27] which is a systematic approach for DA and particularly suited to health policy research. Any documents from the relevant local/regional case study area with sections addressing health inequalities either explicitly stated or implicitly inferred, were included. A list of core documents was chosen, including the local Health and Wellbeing Strategy (Table 1). Subsequently, other documents were identified by snowballing from the core documents and identification by the interviewees. All document types were within scope if produced/covered the period 2018–2023 including documents in the public domain or not, as well as documents concerning a regional, local and neighbourhood level. Attempts were made to include the final version of each document, where possible, otherwise the most up-to-date version available was used.

**Table 1:** List of core documents for the documentary analysis

Regional 1. Integrated Care System (ICS) strategy 2. ICS strategy outlining approach to Long Term Plan (LTP)
Local 3. Health and Wellbeing Strategy 4. Joint Strategic Needs Assessment (JSNA) 5. Director of Public Health (DPH) annual report 6. Clinical Commissioning Group (CCG)* annual report/strategic plan 7. Strategy addressing/outlining approach to Health Inequalities Framework 8. Strategy/policy outlining approach to LTP

*note: CCGs were disbanded in July 2022 and replaced with Integrated Care Systems/Boards

An Excel spreadsheet data extraction tool was adapted with a priori criteria [28] to extract the data. This tool included contextual information (such as authors, target area and document’s purpose). Additionally, all documents were summarised according to a template based on the research aims. Data extraction and summaries were undertaken by CPC and KM.

### Data analysis

Interviews and documents were coded and analysed independently based on a thematic analysis approach [29], managed by NVIVO software [30]. The interviews were analysed using the principles of framework analysis: familiarisation, identifying a thematic framework, indexing, charting, mapping and interpretation [29, 31]. Analysis was conducted using a two-stage approach, the first being an inductive analysis of emergent themes. Secondly, to build knowledge on the case studies’ system approach of reduction of health inequalities, principles of the Action Scales Model was utilised [18]. Comparative case designs can be guided by models, such as the Action Scales Model, to provide an underpinning theoretical model to triangulate and synthesise research findings. Attempts to integrate a case-study with a known framework can result in ‘force-fit’ [21]. Therefore, using a model [18] with seemingly ‘best-fit’ with adaptation enabled us to avoid this. The Action Scales Model provides example questions based on four leverage points (‘events’, ‘structures’, ‘goals’ and ‘beliefs’) which can provide reflection on why the system functions as it does [18].

For the documentary analysis, a combination of both content and thematic analysis as described by Bowen [32] informed by Braun and Clarke’s approach to thematic analysis [29] was used. This type of content analysis does not include the typical quantification but rather a review of the document for pertinent and meaningful passages of text/other data [32]. The interview and documentary findings were viewed as ‘truths’ in themselves, that is, a version of their reality as constructed by individuals [33]. The analysis of each set of themes (with subthemes) from the documentary analysis and interviews were cross-referenced and integrated with each other to provide a cohesive in-depth analysis [34] by generating thematic maps to explore the relationships between the themes. The codes, themes and thematic maps were peer-reviewed continually with regular team meetings. Direct quotes are provided from the interviews and documentary analysis to illustrate key themes. Some quotes from the documents are paraphrased to protect anonymity of the case studies.

## Results

### Interviews

Seventy-eight participants working in the field of health or social care identified through the documentary analysis or snowballing were contacted for interview; forty-six consented to take part. Participant roles were broken down by NHS (*n* = 22), local authority/council (*n* = 13), and voluntary, community and social enterprise (VCSE) (*n* = 11). To protect the anonymity of the participants, their employment titles/status are not disclosed.

### Documentary analysis

Approximately 10 key documents per case study were reviewed (Table 1) except for case study one [23] which was an in-depth pilot case study and 75 documents were reviewed.

### Thematic analysis

Table 2 presents nineteen key themes identified and provides a summary from each case study and whether the theme was identified in the Documentary Analysis (DA) and/or interviews. Gaps in a case study or by interview/DA does not necessarily mean these aspects/issues were not reported/discussed but that they did not emerge during the analysis process as central considerations or were identified as sub-themes only. Moreover, it was originally planned that this work would include a focus around avoidable emergency admissions and questions around this area were included in the interview topic guide. However, as outlined in the previous pilot case study led by Parbery-Clark, ‘addressing health inequalities in avoidable admissions per se was not often explicitly linked by either the interviews or documents [documentary analysis] and is not yet embedded into practice’ [23].

**Table 2:** Main themes by case study, interview and DA

Theme	Case study 1	Case study 2	Case study 3	Case study 4
Understanding the local context	Interviews DA *There was a strong sense that awareness of the wider determinants in health and wellbeing in CS1 have been at the forefront of policy and healthcare for quite some time. The embedding of tackling health inequalities in all sectors was described in the DA and by interviewees.*	Interviews DA *Reporting of higher-than-average levels of death from alcohol*,* smoking and drug misuse. Also*,* an awareness that those from deprived areas of the region are more likely to be high users of healthcare in an unplanned way.*	Interviews DA *Deprivation and inadequate housing were reported to be factors in health inequalities. More lived stories and ‘person journeys’ was said to be vital to help understand the local context better. The COVID-19 pandemic had also impacted the ability to work with communities.*	Interviews DA *The diverse geographical spread of CS4 is recognised. Initiatives are in place to try and engage with communities and to embed co-production strategies into new services/interventions.*
Facilitators of how to tackle health inequalities and improve health and wellbeing	Interviews DA *Many initiatives and strategies were discussed and reported. Major themes underpinning them were collaboration*,* systems working together and communities being at the heart.*	Interviews DA *CS2 appear to be aware of the need of a partnership/ whole system approach*,* and interviewees reported some good practices across sectors. However*,* in the DA*,* it is mostly described as a requirement for something to change in the future to help address health inequalities*	Interviews DA *A wide range of initiatives to support the local population were in place*,* with a strong VCSE sector. The findings from the DA report the use of community-based*,* targeted services. However*,* CS3 are facing several barriers to long-term planning/ solutions for the reduction of HIs.*	Interviews DA *A wide range of approaches to tackling health inequalities are described in the DA and by interviewees*
Looking forward	DA *Opportunities were identified*,* for example*,* the commitment to Marmot principles was considered an opportunity to strengthen future funding bids and additional resource linked to CORE20PLUS5*			
Addressing health inequalities	Interviews *Major themes underpinning the addressing of health inequalities were collaboration working*,* systems working together and communities being at the heart. Interviewees spoke of staff training opportunities and programmes/ initiatives to work collaboratively and share good practice.*			*Interviews: implied in other themes such as ‘intervention facilitators’ and ‘whole system approach’* *DA: identified as a subtheme*
Localised targeting of groups and specific health conditions	Interviews *Reporting of collaboration between sectors and targeted campaigns with communities highlighted established practices*			*DA: identified as a subtheme in ‘understanding the local context’*
Strong partnership /collaborative working	Interviews DA *The importance of partnership and collaborative working across sectors was evident across all aspects of the data. The DA outline phrases such as ‘team approaches’ and ‘system strengths’.*			Interviews *Co-production between systems/the public is highlighted as an important aspect of service use/uptake evaluation.* *DA: implied in the ‘guiding principles’ theme*
Avoidable emergency admissions		Interviews *Interviewees acknowledged that the current A&E set-up/ access to services within the area was not ideal. Services across sectors were under increasing pressure leading to some people going to A&E as they were not able to access other services. Funding was also described as being a major issue*,* with most going to the hospital as opposed to other services.*	Interviews *Much work has been done to support high intensity users of A&E and to support those with addictions*,* multiple deprivation and homelessness with a safe discharge.*	Interviews *Interviewees described strategies in place to try and improve lifestyles*,* keep people well and cared for in their own homes to avoid AEAs.*
Neighbourhood/ community models		Interviews *Work has been ongoing with each neighbourhood developing their own objectives and outcomes based on an understanding of their own inequality issues*		
Access to care/ services		Interviews DA *Many of the issues encountered*,* such as a large housing insecure population*,* and barriers to accessing healthcare were problematic pre-COVID-19*,* however health inequalities in communities post-COVID have been greatly exacerbated creating further challenges. It was acknowledged that CS2 had significant challenges with waiting lists and financial pressures.*	Interviews DA *Access to primary care services has been an ongoing challenge and there is an awareness that many communities/ groups have an unequal access to care and services. The CS3 area is challenged with a significant financial deficit and increasing demand on services.*	Interviews *Access to services for many was challenging due to geographical location*,* rurality and deprivation. Work is ongoing to determine why services might be inaccessible and how they can be addressed*
Workforce	Interviews DA *The main focus was on the workforce being ‘assets’ and the good provision of training and support for staff (especially in the areas of reducing health inequalities).* *Staff capacity*,* recruitment and retention were also highlighted.*	Interviews DA *Main issues highlighted were around staff shortages*,* recruitment and retention.*	Interviews DA *Main issues highlighted were around staff shortages and recruitment challenges*	Interviews *Main issues highlighted were around staff shortages*,* recruitment and retention. Also of concern is the capacity of staff to support the increasing ageing population*
System processes		Interviews *A lack of staff capacity*,* access to follow-up services and financial pressures are impacting the process of the system. A ‘clogging’ of the system was described.*		
Implications of COVID-19 pandemic		Interviews *CS2 was described as being greatly impacted by the COVID-19 pandemic*,* the effects of which were still being faced. However*,* despite the negatives*,* some system working practices improved/ were strengthened during the pandemic*,* which parts of the system are keen to continue.*	Interviews *Access to services post-COVID was challenging*,* it was reported that people were using A&E as a primary care service to compensate for not being able to access the appropriate service.* *The ability to change practices overnight and the way people worked to accommodate need during COVID was reported as a positive aspect.*	
Data and evidence	Interviews *Challenges around accessibility and analysis of data. Local areas to consider the challenges of data sharing across organisations.*	Interviews DA *Active intelligence community but data collection of ethnic diverse groups and qualitative data is lacking/challenging.*	Interviews *Data is not lacking but interpretation of data to business intelligence is challenging. Reluctance across some systems to share data. More qualitative/lived stories needed.*	Interviews *Subtheme in DA* *Community engagement/ involvement/ feedback was reported to be good in some sectors but lacking in others. More is needed to try and diversify the groups who contribute. Some sectors felt frustration at not being able to access certain data sets due to funding or system contract/set-ups.*
Voluntary, community and social enterprise	Interviews *A ‘strong’ VCSE sector is reported but not highlighted as a main theme*	Interviews *A large VCSE sector who are described as being exemplary in tackling health inequalities and helping to ‘plug the gaps’ in health and social care. However*,* a serious lack of funding and investment means many VCSEs are struggling to recruit and maintain staff on short-term contracts and funding*,* leading to pressured teams and restricted services.*	Interviews *The VCSE sector is recognised as an equal partner within the system and plays a strong role within this case study in supporting communities at home. However*,* a lack of thinking and collaboration across some sectors was reported.*	*Acknowledged very briefly in interviews so not highlighted as a theme.* *Lack of knowledge of what VCSE services are available and where to find information about them.*
Future concerns	Interviews *Main concerns were around increased demand on services*,* public confusion of changed healthcare pathways and capacity of workforce.*	Interviews *A lack of national priority of the addressing of preventative measures of health inequalities was reported; too much focus is placed on hospital concerns. Also of concern is the ongoing ‘workforce crisis’.*	Interviews *It was reported that expectations from national government is that CS3 will have recovered from the effects of COVID*,* however it is more likely to be 5–10 years. Finding new solutions going forward for the changed norm is needed rather than trying to ‘fix the current model’.*	Interviews *Future concerns included workforce in rural areas and worsening of health inequalities.*
Supporting local communities/ delivering services in the community			Interviews *Work is ongoing to try and connect more with groups in their locality/ communities. Wellbeing hubs have been set up. A pilot scheme to increase local employment in the domiciliary sector is ongoing.*	Interviews *The main themes focussed on support*,* providing safe spaces and holistic thinking*
Systems working / whole system approach			Interviews *Wellbeing hubs have been set up to connect the community*,* GPs and the VCSE sector. Some VCSE sectors feel that there is some disconnect between sharing of data and silo working.*	Interviews *There was disconnection described between some sectors which impacted on timely referrals/ patient progression and knowledge of other sectors/services.*
Personal responsibility			DA *Findings from the DA placed an emphasis on personal responsibility around lifestyle*,* particularly around diet*,* alcohol and tobacco use. The system wants to empower people to manage their own symptoms and conditions. This was not identified as a theme in the interviews.*	
Guiding principles				Interviews DA *Guiding principles to tackle health inequalities focused on available assets and strengths within communities and families to support quality health and wellbeing for all*

As shown in Table 2, some themes were common across case studies whilst others more prominent in one or two case studies only. A sample of themes are highlighted below with illustrative quotes.

Quotes are labelled by interviewee (Int) or documentary analysis (DA) and case study (CS) number.

#### Understanding the local context

All the case studies demonstrated a good understanding of their local context and the issues that they and their communities were facing in terms of health inequalities and the wider determinants of health. Factors, such as deprivation, lifestyle-related conditions and geographical locations were described:

‘Our population experiences higher-than-average levels of alcohol-related harm, smoking-related deaths, deaths from drug misuse and higher rates of hospitalisation for self-harm.’ (DA_Trust Strategy 2019–2024: CS2).

“It’s poverty. It’s poverty. I mean there’s aging population as well, granted, but I think I we had to pin it onto one, it’s income. People have tough decisions to make based on the amount of money they’ve got in their pocket” (Int: PP10: CS3).

In one case study (CS1), reduction of health inequalities appeared to be well embedded in all areas of their system. Improved engagement with communities was reported by some to be a strategy to try and better understand the wider issues:

*“Part of our remit is to talk to protected characteristic groups*,* minority groups*,* to make services better from their perspective around*,* predominately*,* access*,* but maybe picking up where things aren’t quite working right”* (Int: PP8:CS3).

#### Facilitators of how to tackle health inequalities and improve health and wellbeing

Interviewees were asked to describe what strategies they had in place to address health inequalities. All cases studies were able to describe a range of interventions, plans and strategies that had taken place or were planned, although for two of the cases (CS2, CS3) future planning was reported as challenging whilst they were dealing with current crises.

*“I think finding the capacity within the system to drive change is becoming increasingly difficult. Because there are so many crises*,* and responding to crises*,* that that means it’s difficult to find the time to think about what we need to be doing next and finding that thinking time”* (Int: PP4:CS3).

Support from the VCSE sector was described as invaluable by some and the need for a strong collaborative, joined-up system was apparent. The DA findings also highlighted how each area intended to work towards improving the health and wellbeing of its communities.

‘It is a whole system approach which aims to co-ordinate neighbourhood models of working between health and care and key public services. Recognition that if [we] are to shift the dial on persistent health inequalities in communities [our] model needs to include all key place-based services.’ (DA_Locality Plan Refresh: CS2).

*‘There’s a really strong desire and ethos around understanding that we will only ever solve these problems as a system*,* not by individual organisations or even just part of the system working together. And that feels great.’* (Int: LP3:CS1).

#### Access to care/ services

Two of the case studies acknowledged that access to services/care was currently under severe pressure due to the after-effects of the COVID-19 pandemic, the cost-of-living crisis and system financial deficit (CS2, CS3). Increased demand on services was also an issue. For one case study (CS4) the area’s geographical location impacted on how some communities were able to access services and work was ongoing to try and address inaccessible services.

“MPs [members of parliament] and that kind of thing, when they talk about the Levelling Up agenda, [Levelling up is a UK government mission to challenge, and change unfairness [35]] [they] don’t see rural – rurality – as a metric of poor access, inequalities, that kind of thing (Int: LN3:CS4).

*‘It is important to recognise that our urgent care system is not performing as well as we think it can; that waiting lists for planned care need to be brought down; that financial pressure remains evident across the system*,* that the certainty of funding for social care remains unresolved’* (DA_ approach to LTP: CS2).

#### Data and evidence

Overall, the case study interviewees reported that they had access to adequate data, although some stated that the collection of more qualitative data/real-life stories from people/communities was required. The main issues with data were having the time and capacity to analyse and interpret data to practical intelligence. Also, of frustration for some, was a lack of accessibility of certain data between some sectors or willingness to share data.

“So we’ve done a lot of work trying to make sure that we are all measuring the same things, but it needs resource, it needs software, it needs people to be trained to do the inputting, and it needs the capacity to be able to suck that out and provide some meaningful data” (Int: B3:CS2).

*“I think we have the data we want*,* and we find helpful. I think more qualitative data would be helpful. But it is quite resource intense*,* and it is quite frustrating when we’ve got all these systems*,* we put all this data into*,* and we won’t get what we need out of them. We circumvent the systems. But longer term I hope we won’t be always having to do that”* (Int: PP5:CS3).

#### Voluntary, community and social enterprise

A lack of funding for VCSE and social care was reported as impacting the reach of services. The importance of and the role of the VCSE sector was highlighted as a major theme within the interview data of two case studies (CS2, CS3), with services being described as helping to ‘plug the gaps’ and being recognised as an equal partner in health service operationalisation. Of the key documents analysed, this theme was not so prominent in the DA findings.

“The way the voluntary sector is resourced and still the way we are commissioned, and grant funded mitigates against the interventions being as successful as they should be” (Int: B6: CS2).

*“I think it’s seeing the voluntary community sector as an equal partner and not just a service provider. So it’s not a relationship based on money anymore*,* and it used to be. Absolutely used to be. It’s now a relationship based on outcomes*,* on mutual trust and friendship probably as well”* (Int: PP10: CS3).

#### Future concerns

Main concerns were focused on national priorities of health care, workforce and worsening health inequalities.

“I’m concerned that there isn’t enough focus on it at a national level that it’s the easy thing to ignore, because nobody shouts about it. So, nobody shouts about people dying early, but people shout when there are big queues in A&E, and ambulances are being delayed. They are really important issues, but nobody is shouting about this [health inequalities] as an issue” (Int: B13:CS2).

*“The recovery of that [COVID-19] is five to ten years*,* and yet our population’s expectation*,* and national government’s expectation*,* is that either everything’s fine*,* or we’ve bounced back from COVID*,* or whatever it might be. So*,* I think it’s going to be tough to keep the morale of the workforce buoyant*,* over that period of time”* (Int: PP3:CS3).

#### Personal responsibility

One divergent theme identified was that of personal responsibility and an emphasis on lifestyle which was heavily featured in the DA of one case study (CS3). This was not identified in the interviewee data, so there appeared to be a mismatch between the strategic policy lens and what was happening on the ground in practice.

‘Four behaviours that increase the risks of chronic diseases are poor diet, inactivity, alcohol misuse and smoking tobacco. Risk behaviours remain most prevalent in communities where people are poorest and have lower educational attainment’ (DA_Thrive Report: CS3).

*‘Supporting people to manage their condition(s) to reduce their dependence on professional help’ (DA_Local Plan: CS3).*

In the secondary data analysis, the Action Scales Model [18] provides a deeper level of understanding from the case studies themes of how the system functions and highlights potential areas for action. Table 3 outlines the meanings of the potential leverage levels of the ASM as taken directly from Nobles, et al. [18] and suggested actions.

**Table 3:** The action scales model: potential levers level and actions [18]

	Events	Structures	Goals	Beliefs
What we observe	These are the issues (behaviours and outcomes) that can be observed around us in the modern world and are symptoms which arise from the system functioning as designed (both intentionally and unintentionally).	This relates to the underlying structures and patterns that cause the events to occur. This includes the organisation of the system; the structures, information flows, processes and relationships between parts of the system	These are the goals, targets or ambitions that the system – or parts of the system – is working to achieve. Goals often drive the system to be structured as it is and therefore to work as it does	These are the deeply held beliefs, norms, attitudes and values (i.e., the mindset) of the individuals and organisations within the system. They are the foundations that cause the system to keep functioning as it does and are reflected in the system goals.
Actions at this level	Aim to suppress the immediate event. They do this by reacting quickly to the visible issues – i.e., ‘quick fixes’. Quite often these actions are needed but will not address the underlying issues which cause the issue to arise (i.e., the structures, goals and beliefs).	Aim to reduce the number or severity of the events occurring. They do this by reshaping or redesigning the organisational or relational system structures, and therefore require an understanding for how the system works	Aim to re-orientate the goals that the system is working towards. They do this by changing the beliefs of those people setting the system goals.	Aim to change how individuals and organisations (who influence how the system works) think about the problem. They do this by challenging and changing the deeply held beliefs, norms, attitudes and values within the system

The Action Scales models provides questions across the four ‘observed’ elements; for example, in *events* the researcher is asked to consider ‘the issues or problems that keep arising despite best efforts to rectify them’ and ‘where are intervention efforts targeted’? In *beliefs*, one question asks ‘What beliefs do these people and organisations hold regarding how the system works, and the goals that the system is working towards’? Table 4 reports the potential levers of action identified in the combined systems thinking interview/DA analysis.

**Table 4:** Case study potential levers of action utilising the ASM

	Case study 1	Case study 2	Case study 3	Case study 4
Potential levers of action	• Review use of funding and its sustainability • Review potential of digital innovation but not leaving people behind who are digitally excluded • Availability and use of data to measure impact/progress • Optimise retention and recruitment of workforce	• Funding for and sustainability of VCSE sectors • Optimise recruitment and retention of workforce • Increase collection of qualitative/ lived stories • Further development of the NHS Outcomes Framework and how the data flows within the system and between organisations • Review of system structures/ working post-COVID and structural changes such as ICP/ICS • Address housing/ homelessness issues	• Optimise retention and recruitment of workforce • Increase collection of qualitative data/lived stories/patient voices • Review shared access to patient data across sectors • Review collaborative working/shared vision for all local partners • Allow time and resources for long-term planning • Return to/focus on preventative services/strategies • Reduced emphasis on personal responsibility and lifestyles, alongside a greater emphasis on wider determinants	• Optimise retention and recruitment of workforce • Devise tailored approaches based on specific needs of communities (e.g., rural vs. coastal) • Increase diversity of patient/public involvement/engagement activities • Consider how information from VCSE sectors can be shared/disseminated

Across the studies there are shared identified areas for action; these included recruitment and retention of workforce, collection of/increased diversity of qualitative/lived stories and data flow/sharing. Case studies 2 and 3, which were identified as dealing with challenging aspects of financial deficit and ongoing crises, had a particular focus on the reviewing of system structures, structural changes and long-term planning.

## Discussion

Using case-study methodology, our study aimed to explore and understand the challenges, opportunities and drivers for local areas addressing health inequalities in four areas of England.

Whilst each case study area was aware of the importance of working as a system across sectors towards the reduction of health inequalities, contextual factors within cases are likely to affect the success in doing so. Highlighted across cases was the prominence of operating in collaboration with the voluntary sector and communities as an integral aspect of working to reduce health inequalities, however, the evidence around whether collaborations between local health care and non-health care improves health outcomes has been reported as mixed [36]. The embedding of a community/neighbourhood model and the collection of real-life stories/data from diverse communities, was described by some as a future goal [36]. Findings from a systematic review exploring community engagement in improving the health of disadvantaged populations [37] show that the majority of community engagement participants were empowered and improved their social networking and self-efficacy skills. However, the authors also conclude that any unmet needs identified through the community engagement can also lead to participants feeling dispirited. This is especially so among ethnically marginalised and indigenous communities, resulting in poor individual health outcomes [37]. Therefore, it is imperative that community engagement activities, as highlighted by the ASM, are supported by action [18].

The vital role of the VCSE sector was reported by many, with services described as helping to ‘plug the gaps’. However, a lack of sustainable funding and, at times, lack of recognition and integration within a system was apparent. Whilst many of the interviewees spoke of the importance of the VCSE sector contributions, it was not identified as a main theme in the key documents analysed as part of the documentary analysis; this perhaps illustrates a mismatch of the strategic vision for local areas outlined in these documents and the reality of what is happening ‘on the ground’. It is important to understand and take into account micro level issues and factors that influence those working ‘on the ground’ who have to interpret and action policy [38]. Interviewees working in the VCSE sector described how they had scope to work more flexibly in their roles, leading to more responsive services. This finding was also described in a recent qualitative study conducted with VCSE staff in Northern England; staff reported that, during the COVID-19 pandemic, they were required to be agile and adaptable and felt frustrated that their intimate knowledge of local communities was not often acknowledged by statutory sectors [39]. Whilst the COVID-19 pandemic increased intersectoral collaboration; decision making between actors has been reported to be unequal within systems [6]. A further study by Carpenter et al., also exploring the role of VCSE sectors during the COVID-19 pandemic, reported that if the VCSE sector is to have a key role within an integrated care system, the issue around partnership working needs to be addressed, especially for smaller grassroots organisations which might be overlooked [40].

The effects of the COVID-19 pandemic were keenly felt in two of the case studies in particular. The negative effects related to increased demand on services, changed care pathways and confusion by the public on how best to access care. Access to certain services, such as management of people with diabetes, hypertension or multi-morbidities, has been reported as being significantly impacted as a result of COVID-19 [41]. However, positive impacts of COVID-19 were also reported by several sectors across the case studies, such as more responsive ways of working and collaborative practices within the system, which case studies were keen to preserve going forward.

Workforce recruitment and retention was a concern in all sectors, with ‘burnout’ and low morale an issue for some areas long before the COVID-19 pandemic which exacerbated the issue. Some of the case studies are addressing the problem with recruitment drives and initiatives to ‘grow’ local workforce in the care sector. However, whilst areas can strive to improve the situation locally, as reported by a recent Lancet Health Policy paper on the UK health and care workforce, there is an urgent need on a national level to address the current workforce challenges to secure a sustainable and fit-for-purpose health and care workforce going forward post-COVID-19 [42]. A 2021 post-COVID-19 pandemic Lancet commissions paper [43] outlining priorities for action on the future of the NHS, aligns with findings from the current study. Identified areas for action such as social care funding, workforce planning, improving population health and strengthening integration [43] are comparable.

The challenge however is that, despite policy, guidance and frameworks [5], health leaders feel they lack skills and knowledge to reduce health inequalities [5]. Nonetheless, as demonstrated by this case study project, it was apparent that two of the case study systems (CS1, CS4) appeared to have more resilience in riding out and recovering from the COVID-19 pandemic. These case studies demonstrated shared ambition across systems, strong partnership working, an understanding of how the system works and putting their communities at the heart of the system. Having a strong footing may have helped with unexpected challenges, such as COVID-19. The other two case studies (CS2, CS3) acknowledged difficulties with increasing financial pressures and ongoing crises; interview participants reported how constantly having to deal with one crisis to the next (described by the ASM as constantly responding to events with quick fixes) [18] impeded capacity and thinking to reflect and plan for longer term. The Office for Health Improvement and Disparities (OHID) emphasise that addressing health disparities and inequalities requires collective efforts from health professionals, practitioners, leaders, and community workers [1]. However, to have a shared ambition and strong partnership working, systems need to change how individuals and organisations (who influence how the system works) think about the problem [18]. As described by Nobles et al., [18] they do this by challenging and changing the deeply held beliefs, norms, attitudes and values within the system.

### Strengths and limitations

It should be noted that the findings/conclusions from the case studies are context specific and based on the interviews of those particular stakeholders/sectors who agreed to take part. Likewise, the DA analysis was a high-level analysis determining the key themes from core documents. Therefore, the conclusions/recommendations are based on the data available to the researcher at the time. This study uses a methodologically rigorous qualitative case-study approach to investigate inequality in complex systems [44–46]. Furthermore, it is the first time to our knowledge, that that the Action Scales Model [18] has been adapted from its initial conception to support the evaluation of case study data focusing on addressing health inequalities a complex multi-agency system.

A recognised limitation is that the themes and leverage points identified from the case studies analysis are unlikely to represent an exhaustive list of the key elements requiring attention. However, they do represent the key themes that emerged using a robust methodological process. Attempts to integrate a case-study with a known framework can result in ‘force-fit’ [21]. Therefore using a model [18] with seemingly ‘best-fit’ with adaptation enabled us to avoid this. However, it should be noted that the strengths and challenges as described in the ASM model ‘beliefs’ section (Table 3) are an implicit interpretation and not explicitly reported as beliefs.

Moreover, the use of snowball sampling as a method may have limited the scope of potential interviewees, and not everyone approached agreed to be interviewed.

## Conclusions

This comparative case study makes a crucial contribution in the examination of the challenges, opportunities and drivers in health systems and the addressing of health inequalities in their local areas. The combined interview and documentary analysis findings provided rich insights of local systems’ documented strategies, plans and what is happening ‘on the ground’. Health systems having a shared vision, strong partnerships, understanding the system, and putting people and communities at the heart of decision making, appear to foster system resilience despite the many challenges currently being faced post-COVID 19.

## Supplementary Information

Supplementary Material 1.

## Data Availability

No datasets were generated or analysed during the current study.

## Abbreviations

NHS: National Health Service
OHID: Office for Health Improvement and Disparities
VCSE: Voluntary, Community and Social Enterprise
DA: Documentary Analysis
ICS: Integrated Care System
LTP: Long Term Plan
JSNA: Joint Strategic Needs Assessment
CCG: Clinical Commissioning Group
ASM: Action Scales Model
