# Supplementary material for: Understanding the challenges, opportunities, and drivers to addressing health inequalities within local health systems: the UNFAIR case study qualitative project
Source: BMC Health Serv Res. 2025 Jun 6;25:810. doi: 10.1186/s12913-025-12956-7 (PMC12143079; doi:10.1186/s12913-025-12956-7)
Supplement: Supplementary file 1 — Supplementary Material 1. [file 12913_2025_12956_MOESM1_ESM.docx]

Introduction and Welcome

- Consent – e-consent returned?
- To the researcher
- Aims of the project
  - To explore and develop an understanding of how local areas address inequalities with a focus on avoidable emergency admissions.
  - To co-produce with patients, the public and health professionals, practical learning to help local area decision makers reduce inequalities.
- Aims of the interview
  - Get an overview of their role generally, then in relation to Health inequalities (HI) and avoidable emergency admissions (AEA) in particular
  - Identify key people in local area with a role in AEA
  - Identify key interventions around reducing HI and AEA

What is your role?

- Time in post
- Background

To what extent are HI/AEA a priority in your work

- Expand / give examples
- Collection of data?
  - Where is the data sourced?
  - Type of data
  - Is the data useful?
  - Enough evidence?
  - How is data used?
- Who do you link in with?
  - Meetings/minutes/reports?
  - Terms of reference for groups?
- What factors affect HI and AEA in your area?
- What HI/AEA work have you been involved with over the last 2-5 years?

In terms of HI/AEA what are the key interventions/programmes in your area

- Who is involved?
  - Service users/patient/public voices?
  - How is information collected/collated?
  - Representative?
- Staff training?
- Any documents/ reports?
  - Can they screen share/put links in chat?
  - Can they share terms of reference?
  - Can they share reports/minutes of meetings?
- How successful have they been?
- What improvements / changes have you seen?
- What makes for successful interventions in this area?
- What are the key barriers?
- Do you have any future concerns?

Anything you want to tell me about HI and AEA in this area that we haven’t covered?

If time allows ask interviewee (if they haven’t already done so) to name/share their 3 contacts/groups and documents/websites etc. This can be in the form of screen sharing which will be captured in the recording or paste links in chat.

To make a record on a spreadsheet of key people identified, their role – to then go on and find out contact details

Also record on same spreadsheet key documents and then find copies and link/save on document. One of these for each case study area
